# Supplementary material for: A painful journey to antivenom: The therapeutic itinerary of snakebite patients in the Brazilian Amazon (The QUALISnake Study)
Source: PLoS Negl Trop Dis. 2021 Mar 4;15(3):e0009245. doi: 10.1371/journal.pntd.0009245 (PMC7963098; doi:10.1371/journal.pntd.0009245)
Supplement: S1 Text — (DOCX) [file pntd.0009245.s001.docx]

**** *Interview_1

‘The week that started on a Monday’ was the week of the “milk festival”, and the mayor gives up the schools for people who have relatives who live nearby to sleep and stay in schools. This whole week I was off. The mayor anticipated our payment due to the holiday, so I took the opportunity to go to the smallholding to finish getting lumber to fence the land that is only fenced on one side. My husband also went to cut wood because he has a chainsaw. We also took a young lad to help us and made up a small parcel of food supplies to take.

That day we went on Thursday to return on Monday. When he went out on the farmland on Friday morning, I got up, made the breakfast, then we went out a hundred meters outside the house where my husband had cut down a tree for lumber and make the fence, but that land's all cleared because my husband clears everything so he can work. Cleared with a rake, right and everything is very clear, except that there is a snake in the hole of a piece of wood. Then, around nine in the morning, I left to go for pee because the boy who was with my husband is not my relative, right? So I moved away, closer to the forest, because there is no bathroom in the house. I peed and when I came back I felt like someone had taken a stick and poked my leg. I was wearing boots, but they are short and the snake bit above the boot. Then I walked a little further and stopped. That's when I felt the blood coming out. I looked up and I felt like I was going to pass out, like my vision was blurred. Then when I looked back I saw the snake. It was dark with brown spots, then I started to get nervous and I thought to myself “oh Jesus, calm me down”. I started to taste blood in my mouth, I took the boot off my foot, and that's when I saw the blood coming out. Then, I called the young lad, screaming loudly for him to get my husband who was cutting wood with a chainsaw. Then he came to me and I said "Oh love, a snake bit me" and he said "Where, Sida?" and I told him “Right here, close to the forest”. Then I told the young lad that helps us to get the motorbike and take me to the town of Autazes.

My husband came and actually saw the snake, but it slithered away from him. I said to him "Leave that snake there and come with me because I'm feeling sick". I started to feel like my leg was heavy and it made me feel sick, just like I was going to pass out, you know? My vision darkened. Whenever I go to the site, I have a bag where I put my ID, so that in an emergency like this I am prepared. So I just got my bag from the house. The boy had already gone to get the bike and my husband said "Calm down love, the snake is not venomous", then I said "I am calm, but it is no use lying because the snake is venomous. Blood is coming out of my mouth and I have a very bad taste”. Then I said “take the key to the house because the boy will take me. Leave it there! Don't worry about that, you should worry about me!”. So we got out on the bike, but from where we were, it's three kilometers to the nearest community and from that community to the city, it's twenty-five kilometers.

When we on the main highway, already in the community, I felt my vision getting darker. I asked him to take me right to the community's accident and emergency riverside ambulance service because on the back of the bike I felt that I couldn't take it anymore and this community, called Sampaio, is an indigenous community where there is a health post, school and ambulance. I remembered that I could get help there and as the bike was already old and the boy couldn't go fast, I could fall off the bike or pass out, so I asked to go to the community. So he went to the community and, when he got there, the ambulance was already leaving with a little girl who had fallen and had her arm in a sling because she had broken her arm. I immediately asked for help from the driver “Hey! A snake bit me, help!” then he said "So get in quickly and don't waste time, because here you’ll only get antibiotics and painkillers, we don't have what you need", and I just got in the ambulance. When I got close to the city, I told the driver that when he arrived he should ask for help because I might not be able to walk and soon after I passed out. When I woke up I was already in the infirmary, I had oxygen because my blood pressure had dropped and my leg was already swollen. They had already applied antivenom and performed the procedures. They applied the antivenom and an hour later they collected my blood to see if it was clotting and saw that it [the antivenom] was not working because my blood was not clotting. Around 12:30, they called my husband and said they were going to take me to Manaus because the antivenom they gave me was not working and the snake's venom had been very strong. They had already made the request because now there is this system that automatically calls the ambulance via the system, right?

From Autazes I came to Careiro by ambulance some fifty miles overland. In Careiro Castanho I crossed to the port of Ceasa in the small motor boat and here in Manaus there was already an ambulance waiting for me. I arrived the same day! Where I live there is no help when there are accidents. The people who live the deep in the countryside are the ones who take home remedies, but I don't know of any. I heard it said once that when you get bitten, you should take the snake's guts out and put it on the bite because it draws the venom out and you can also use a tourniquet, but I don't know if it works or not.

I haven't taken anything before, no home remedies. My husband wanted to tie my leg up but I didn't let him. I didn’t let it be tied up because old people say to tie it up so that the venom doesn’t spread, but that’s old people’s talk, right? But many of these things I don’t believe in because I’ve seen many people do it and it didn’t have an effect, they’ve died, including even my uncle who was bitten by a snake. But in his case, the medical assistance took a while, he lost his leg and he had tied everything up. I remembered that when you tie the limb up, the blood doesn't circulate, so I didn't tie it and the doctor confirmed here that you only have to apply the dose of the anti-venom.

The biggest difficulty is the distance, because if I had a plane it would be faster, but I don’t. On the way from the smallholding to the city there is a stretch of about fifteen kilometers that is bumpy and the road is not good. The bike is not always available and I am often alone and my husband comes to pick me up the next day. The nearest telephone signal is one kilometer away from the smallholding.

The complication I had was the abscess that needed to be drained, but it was just that.

**** * Interview_2

I live in a community near Careiro Castanho. There, on the way to the port, the insect bit me. I came home and, as I didn't know if it was serious, I decided to go the hospital. Within an hour I left the house, rode an hour in a canoe with a small engine, but the hard part was to catch the buses which is difficult. As I said, it's an hour or two just waiting for them.

I traveled to Careiro and from there I boarded a boat-bus, which is a struggle because when the tickets are sold out, we have to wait and risk here getting late at the Tropical Foundation.

I work in the fields, planting corn, sometimes potatoes, sometimes yams. When the accident happened, I was getting ready to go to the fields. I live of the fish I catch and had nothing to eat, so I went to repair a net that I had arranged to catch some fish and bring them to my wife. Where I live, it’s not always time to plant, we have to plant in the [seasonal] low tide to harvest in the flood [season]. I was going to catch the fish so that I could go harvesting, because the farm business is a sacrifice, you have to cut down trees, you have to drill, burn it every now and then. Then you plant and you have to go weeding that field and take care until you harvest. This is not something you put there and leave.

I went to get the net and on the way home the snake bit me near a river. Near this river there is a community where one hundred and ninety residents live, called the Lago do Mira community. Right after the bite I went home and had some herbal tea, it's a tea made from a leaf, which they said was good and that's what I drank. It was a tea that we prepare by putting the leaf in the water and then boil or else put it in a blender and drink the juice. Either way works.

Several people told me it is good, everyone there already knows that it works. You plant what you need so that when a snake bites you, you can drink tea. In reality it is a faith cure because when you are out there in the jungle and you don't have this healthcare like you have here in the city, then we use the resources we have there. I only drank this tea before coming to the Tropical Foundation.

I was barefoot, because I was close to home. I saw the snake only after it had bitten me. I hit it with a stick and smashed it. I drank tea to be able to endure the trip and I waited an hour or so for it to take effect so I could endure the trip and my daughter-in-law got me ready. I came in a small boat with a small outboard engine, until I reached the main road. We got to the road at highway marker fifty-four, and we waited for the bus to go to Careiro da Várzea where we took the small motor boat there. Everything took too long. When I arrived at Careiro, it was almost noon. I came with my daughter-in-law and my grandson and in Careiro my son-in-law went to meet me there, he lives here in Manaus. Arriving here in Manaus, he ordered an Uber so we could get here to the Tropical faster. I knew I had to come here because I had already treated myself here before, in 2003. This is the second accident I’ve had treated here, but in total there were nine, only this time it was on the left leg, on the heel. The previous bite was on the right leg here in the calf. They took me to the 28 de Agosto hospital in 2003. There the doctor cut my whole leg because when I arrived it was already very bad. That's what they say, because when I arrived, at the time, I was unconscious. The other time, on the trip they brought me, they took me to the 28 de Agosto hospital, there I did exams and cut my leg [open]. Then they sent me to Tropical Foundation where I was unconscious for about fifteen days and in total it was a month and a week of treatment. When I woke up I didn't even know where I was. At the time, I didn't drink anything because I didn’t know about the tea yet. This time I was at the same lake, but it was on the other shore where it bit me. I was going over a tree trunk when the snake came forward and bit me. When I saw it, I remembered my rosary and when I turned to get the rosary I fell over backwards, since I was already anesthetized. Then it went back like that and my son said: “Dad, I'm going to kill it!” and I replied: “Don't! It already bit me and it will bite you too!” Then it left and three days after my nephew went there and managed to kill the snake. This snake that bit me last time was one species and this time it was a different one. The health agent taught me about this little plant to make this tea, and she managed to get me a seedling to plant.

The whole difficulty is the distance, because you take a canoe with a small outboard motor, buses and a small motor boat, all to get here. Every day I do this routine, because we live on it. If we fished and caught a lot of fish, we could go days without fishing, but you go and get that little bit that you can only eat there on the same day. I don't even like it anymore because I'm always sick of eating “Piaba”. The hunting is difficult because my sight isn’t good anymore. At home now there are four people including me, but it is usually just me who works because my wife who is regularly ill is usually here in Manaus getting treatment and my children also stay in Manaus with her, so in Careiro there is usually only me. Most of the time I am alone, but luckily this time I was not alone or it would have been difficult. Life in the countryside is good, but it is good and bad because it is difficult. There are places that have a telephone signal and others that do not, there is power every other day or a week (laughs) but that's how difficult it is. I have many neighbors at least, right, because we live close by, but it’s not always that the closest neighbor to me is at home, so if something happens and you are alone you would have to be lucky to find a neighbor at home too. I could even have one at home, but a lot further away. Some live off the fields, others off livestock raising, some plant closer by, others plant further away. I arrived last, so I plant in the wetland, because if I plant in September, in April I have to harvest. Thus, the life of the small farmer is not easy. Things happen and we learn. Because what I went through here and not only this I do not wish even my worst enemy. This penultimate time I almost lost my leg, right, so we learn.

**** * Interview_3

I went to school, because I study at night, but our CPU had burned out and there was no class, so my colleague who lives near the school invited me to go to his house and wait for the boat until about 10 pm, because the class starts at 7 pm and ends at 10 pm. As it was early, we went there by the trail. We were using a flashlight and when we got to a certain part, where there was a fence between one field and another, I went to the fence to open the gate, that's when I felt the bite. My school is in another community which is not the one I live in, that is the São Francisco community. When I felt the bite, I shined the flashlight down and saw the snake stuck to my foot. Then I told my friend that the snake had bitten me and shook my foot, it retreated and I just felt the blood spurt. Then he went to a woman's house nearby, took a stick and killed the snake. My foot started to bleed. At the time I didn't feel anything, so I stayed at the woman's house nearby while he killed the snake and he tied a shirt here on my leg so the venom wouldn't rise, just above the bite. He cut off the snake’s tail, right, because they say it’s good. Soon after it bit me, I was in a lot of pain. He cut off its tail and put it on top of the bite and that was what made the pain go away at the time, because they say it draws the venom from the bite. The bite happened at about 8 pm and the boat had not yet arrived. The pain died down and my colleague had a canoe with a small motor in the house I stayed at, so my colleague went to drop me off at Mom’s. My mom was at church and I just asked him to help me get over to where mom was. Then we went after another canoe with a small engine to take me to the town of Manacapuru. Using a canoe with a small engine, it took five minutes to get to Mom’s. Then we went after another canoe with a small engine but with more power to take me to Manacapuru, but the owners did not want to lend it to us out of fear of the drug traffickers on the Solimões River. Then we went to the health care worker, but the accident and emergency riverside ambulance service wasn’t working to bring me here. Then, they just gave me another home remedy. We even went to the health care worker, but the accident and emergency riverside ambulance service wasn’t working, and could not be called and it was already 9 pm. They just gave me a few pills, five pills from the home remedy, which was how I managed to wait it out until the time the small motorboat arrived around four in the morning the next day.

I had heard of this medicine that was good for snakebite, but I had never seen anyone take it. I was told it was good for snakebite. When we arrived in Manacapuru, at the hospital, they put something in the vein, an access, they did not give me antivenom, they immediately gave me the medicine to stop the venom. That was about six o'clock. We stayed there waiting, that's when they took my blood and then they told me that I would come here to Manaus. We came here by ambulance, me and another woman who was feeling sick. We arrived at this hospital at about 11:30 pm. It was the first time coming here that I heard about this accident like that. I untied the shirt right after I took the home remedy. The pain did not take long because my friend put the snake's tail and that was how the pain went away, it was as if it had drawn the pain out.

**** * Interview_4

We have a path we use to get to the lake to fish. We went down this path around five-thirty in the morning, we walked slowly and when we were near the lake, at about 6 in the morning I just felt the snake bite me. This lake is close to home, about fifteen minutes on foot. When it bit me, I told my brother who was right behind me and at the time we couldn't kill it. My brother ran ahead to put the canoe with a small engine that we have in the water, a little engine fifteen HP, then it was forty minutes until we reached Manacapuru. I didn’t put anything on the bite, I didn’t put anything on it or over it because it’s not recommended, is it? I heard about it from the doctors, because my brother was bitten once and what he drank was condensed milk and that's what I drank too. My brother said it helps to stop the effect of the venom, so I took it before getting in the boat because there was a grocery store near the river and my brother got some for me.

My brother-in-law was already waiting for me in Manacapuru with the car at the marina. We went to the city hospital and I took the medication just to make the pain go away and I was waiting for the proper medicine for the venom. After a few hours they told me that I didn't have the anti-venom and that I would have to come to Manaus. They brought me here in the ambulance at about 11:30 am. I knew more or less what to do because my brother had already suffered this type of snakebite before and with me this was the second, but the first bite did not swell up and did not hurt like this. I spent the night at the hospital in Manacapuru and they discharged me and sent me home, because it didn't hurt, it didn't swell. I had the anti-venom for the venom only because I came here because I didn't have the anti-venom and at the time of the first bite I had, just not that time. I didn't have much difficulty because I already had all the transport there and was very careful even on the rush because this is not something to waste time over is it? At the time, I was with my two brothers, one went in front to help me and the other went to warn the family.

**** * Interview_5

We were going to hunt for an ant that is a food in our culture. It is found in a hole in the earth, then we take a stick from the palm itself and put it as far as the ant is. Then we take the ants out and put them in a bowl of water. It was about 9 o’clock and we were on the way to get to where the ants are, but on the trail it looked like the animal was waiting for me. That's when I felt the bite like an ant's bite. It was on the trail. I was with my brothers at the time, I killed the snake and threw it away, I took a vine from the forest, we cut about a meter in length and drank the juice. It has a slightly sweet taste. Then I came home straight away, poured gasoline over the bite and went to the riverside, where the health care worker took me to the area's health center. I got home at about 10 o'clock and got to the health center at about 11 o'clock. To reach the health center we went by canoe with a small motor. There, I had an injection to relieve the pain and they sent me by small motor boat to the hospital in Maués, but there were also no resources there because I had no anti-venom and I was hospitalized for only one night. I arrived in Maués at about 3 o'clock in the afternoon and stayed there at the hospital until about 7 o'clock the next day. I came by plane and when I arrived here at the airport there was an ambulance from the Indigenous Health Service waiting for me and they brought me here to this hospital. If I were to come by boat it would be a day and a half to get here. It took so long because there was only a plane leaving the next day. When I arrived here at the hospital, it was about forty minutes of flight and I arrived here at eight in the morning. This was the first time this happened to me but, with other relatives, this type of accident has already happened. I just poured gasoline over the bite. I didn't do anything else. It was my father who said that using the vine was good, that everyone knows, but when this happens to us because of panic, we sometimes forget all the medicine that our ancestors passed on to us. This one about gasoline was another relative of another tribe who taught us to pour it on the snake bite. We only spill gasoline on top of it because of the pain, you know?

When these accidents happen and we know the medicine to use, we make the medicine we know right there in the woods and from there we make the direct referral to the health center. In these cases of the snake, we just go straight to the health center, but other lesser accidents, if they are not snakebite, we stay with the tribe.

This is the first time I have taken this, I was even asked if I was allergic to anything but I don't know. I just know that this is dangerous because my father says that with a snake accident if it doesn’t kill you, it will cripple you. If I don't take medicine, my leg could become inflamed and I might lose my leg, or I was told that if it darkens my vision and I might lose it, so that was my fear, but what I felt was only pain and swelling.

**** * Interview_6

Where I live there was a power outage yesterday so I went to the grocery store at night to buy candles and some incense that we put out to scare mosquitoes away in the morning. On the way to the tavern, I got in the car and went to the highway, but when the car stopped and I opened the door to get out, the snake was crossing the highway and I didn't see that it was close to the wheel and I stepped on it. This car belongs to my cousin who was driving and I was on the passenger side. When he stopped, I was already opening the door and getting out. There was a colleague of mine coming from behind on a motorcycle and he shouted that there was a snake there, but I didn't hear him. He thought that the boy would run it over, but no. We didn't see it in the dark, with the power out. I stepped on it, but I don't know where it was because it just turned and bit me. That was about nine o'clock at night when it happened. The boys went out shining the light of the cell phone and I felt just a bite at the time. We only saw it leaving. When I looked at my leg it was bleeding. I didn't even feel much pain, just a little prick. After about three or four minutes, I felt like my blood pressure was dropping, making me dizzy and my heart racing. And I was afraid of dying. Then we went home soon. I remembered that if we squeeze a cloth and contain the venom, it wouldn't pass through the body, right. At the time, I tied my leg up and a lot of people were helping. I tightened it on one side and another person on the other side of the knee, holding it. I told my sister that I was going to Manaus and she went to call an ambulance, because there is an ambulance in the community. She went by motorcycle to another community a kilometer away to call the ambulance. The ambulance man came and went to another community called Santa Luzia to get a nurse to accompany us. I live in Nova Aliança and the ambulance was at the ambulance man's house but the nurse lives in the other community. It took us over an hour to get to the Ceasa port. Arriving at the port of Ceasa, we spent almost an hour also waiting for the small motor boat, because it was on the other side and we had to wait for it to cross back over the river to pick us up. We had to call the small motorboat to cross back because otherwise it would take too long. Then we crossed and there was no ambulance when we got there, we had to wait more than half an hour to get to the ambulance to come and I screamed inside the small motor boat, along with the ambulance driver, the nurse and my friend. My leg, still tied up, was swollen and all purple.

When the ambulance brought us here, it was almost one o'clock in the morning. And I was afraid of dying, my heart felt tight and was beating very hard and my blood pressure was going down and I was thinking "Jesus I'm going to die. Don't let me die". Then I felt the pain even more, the dizziness was only at the beginning and my heart was racing. And this pain that doesn’t stop and there’s a time that it seems my whole leg is going to burst apart, and I feel like I’m all numb. It really hurts! As if it was going to burst apart. The idea of ​​tying it up was the neighbor’s idea. He said it was good. It is a myth of the interior. They say that the snake has venom and if you tie it off, the venom does not flow up very fast and does not kill the person. They say that if the venom gets in the heart it kills. As I was afraid, they started to tighten the tourniquet, but the doctor told me off. But, in moments of despair, we don't even know what to do, because, at the time, the most important thing is my life.

I decided to go immediately to the hospital. I went into despair saying: “I want to leave, I want to leave” and the ambulance didn’t seem to be coming and there was no way to come right away. Thankfully, the guy on the bike soon went home with me and was already helping to call the ambulance. The boys still went to kill the snake with stone, but I didn't even want to know.

I work with agriculture but this never happened before. I already found three snakes inside my house, but they never bit me. One in the wardrobe, another one I almost touched and another one is adopted and lives on top of the hose, but it doesn't bite, every day it stays on a branch. I went to get a glass to drink water and the snake was already on the side of the glass and I didn't even see it, but it didn't bite me. That day of the bite, we were celebrating my grandson's birthday, he had even had a few beers. Then the power went off, everyone stopped and I went to get these candles. At the moment I am here alone, waiting for someone to come and help me take a shower, because I am the way I arrived, because I was all dirty except that my leg hurts so much and everything was so fast that I haven't even showered yet.

**** * Interview_7

I was at the smallholding at highway marker thirty-eight on the AM 010 highway. I was coming back to Manaus when it bit me. It was six hundred or eight hundred meters away from the car because the track was a mess due to the storm that had and ruined our track. So I went to clear it and it didn't help, so I left. I came in sandals because I couldn't take wearings boots anymore. I left the car far away because there was no way to get to the smallholding due to the state of the track. On the way back the snake bit me. I go to the smallholding more for leisure, for a walk and I came with my brother and my sister-in-law and I think I stepped on it, then when it bit me I jumped and looked at it, then it bit me again in the same place and I just saw the blood running and bleeding, then I yelled for my brother and that was when he came running and I said: "Kill this snake because it bit me!". It was about two o'clock in the afternoon. With that, we left there and he killed the snake and I went ahead. I got in the car and started the car, feeling horrible pain, so much so that I was screaming and bleeding from my foot, but I kept walking. I got in and started the car, I left it ready to go, but it was my brother who brought the car, who came driving. At the time I just felt a lot of pain and it started to swell up to the knee, then I tied a rope here (on the thigh) so the venom wouldn't rise. I kept it very tight so that when I got here the leg was almost purple. I knew I had to come here because as I live close by in the Alvorada neighborhood. I already knew that this is where these snakebites are treated. The snake bit me around two o’clock and I walked for another fifteen minutes and we arrived here at about twenty past three in the afternoon. The only thing I did was the tourniquet, we just got in the car and left. This is the first time this has happened to me and, knowing that this business is serious, very serious, I came straight here. One difficulty was the traffic. Here they applied the anti-venom through the vein.

**** * Interview_8

I was working at the time, it was about ten o'clock in the morning, I was in the fields because I wanted to start a banana plantation and I was barefoot. I went to cut a thick stick and when I put my right foot in front of it like this to support me, it was when I stepped on it [the snake]. It was under a branch. I hadn't seen hit and when I touched it, it bit me. I saw it, but I couldn't kill it - I was going to kill it to bring it here. Then I went home and told my mother and sister that live with me. My sister is a little bit careful like me, but sometimes I don't treat my illnesses. I took a home remedy called “thirty-three”, which is a pill with a liquid, and I took fifteen pills. I took about ten to fifteen or so and I stayed there until about two-thirty, for three hours, to see if it would swell up because it didn't hurt. I felt nothing, just the bleeding at the site of the bite. I came from there at about four o’clock because my foot was already swelling. There is a guy who works with tourism there and I got a ride with him to come straight here. I came by small motor boat in an hour and a half and, here in the port, I already had an ambulance waiting for me because the lad who brought me here called the Tropical Foundation in advance. In my town, I went to the health care worker, but she just washed my foot with alcohol and put iodine on it, because where I live it is still isolated from everything and then I went to this other community, Tumbiras, to see if I could get a boat to come to Manaus. There, there are health workers who are trained to measure pressure, these things, you know, but she only cleaned my foot and put the iodine on it. It is twenty minutes to Tumbiras where I went by canoe with a small motor and, as I had an acquaintance who works here, the boy who brought me called her at the hospital, and she provided the ambulance. But I only came because it was swelling. I arrived at the port at about ten past six in the morning and at the hospital at about seven. I only took the home remedy, because to my knowledge, which is the knowledge of my late father, we never tie the limb off when a snake bites, because if we tie it off, it will swell and when you release it, with the pressure of the injection, the venom rises all at once, because the blood spreads right? Because the blood rises and the venom spreads, that's why I didn't tie it off - I just took the home remedy.

I had another accident before this one, about twenty-five years ago, but I stayed there [in the countryside]. At the time I took a herbal remedy that is used specifically for the venom. I don't remember the name of the herb, but I’m sure that it works against the venom. At the time of the accident, it was just me and another guy who didn't pass out because I didn't tell him at the time. He's afraid because he's been bitten before.

**** * Interview_9

It happened like this: I was in a rehab clinic and went to do a job carrying some wood. This clinic is on the Pau-rosa track, near Vila São Sebastião. I was close to home, about five hundred meters, and I only saw it when it was going into the woods. It bit me on my little toe of the left foot. I just felt the leg going numb and a little bleeding, just a little bit on the toe, but really just a little bit. In a matter of minutes, it swelled up and was like a balloon up to my knee. I was lying in a hammock and the lads wanted to bring me here so I said: "Boys, this isn’t going to be much, just put a dressing on it". So I just came here the next day. The accident happened on Thursday at ten o'clock in the morning and we arrived the next day at three o'clock in the afternoon. I didn't eat anything, just breakfast and food. I put this paint spray on top of the bite (laughs). My leg continued to swell and I felt dizzy and had a headache, I couldn't walk anymore and they had to carry me here.

First I came by canoe with a small motor to the Davi marina at Ponta Negra, from there we came by car to the Tropical Foundation, in the clinic’s car. It was the pastor who knew he had to come here, that's why I came. It must have happened there before. You can come in many ways, but I came by water. It doesn't take more than half an hour on a small motor boat. In the car to here, it's about ten minutes without traffic. Here I've been taking this stuff and I got sick with it and also with some morphine. It took really long because I didn't want to come. I told the leader there that I had been bitten by a snake and he said: “Nonsense! You are walking normally, look! ”. That was when lunch time came and they had to carry me so I could eat. They carried me from the clinic to the car, where they have to go down a ravine, and on the river bank they carried me onto the small motor boat. All the way I was carried and when I got here they got me in a wheelchair. I decided to come because the leg started to swell and hurt, it was like a balloon and the guys even wanted to play ball with it (laughs).

**** * Interview_10

I was hunting in the low grass beside the stream when I stepped on it. This was the first time that an accident happened there. I was about thirty minutes from home. At the time, I felt pain and my heart beat faster, but what was more intense was the pain at the site of the bite and the headache. At the time, my brother was with me. We just took the canoe and we went home. I didn't put anything on the bite. I just came home - about half an hour in a canoe. Arriving home, we got the canoe with a small engine. It is a very difficult moment, because in the countryside we have to move fast because there is nothing there and this was the second time [this had happened to me], so I already kind of knew what I had to do. The other time, we got it treated in Manacapuru. As we had transportation at the time, we took the canoe with a small motor to Manacapuru which took an hour or an hour and twenty minutes. We went to the Lázaro Reis hospital in the city, but they said that there was no anti-venom there and that we had to come to Manaus. That day, the accident happened about six o'clock in the morning and we were already in Manacapuru by around ten o'clock, but there was no ambulance there, because it was here in Manaus. We waited for it to come back. When it came back, we came here to Manaus. It was already nine o'clock at night. Here, I took the medicine to prepare me to take anti-venom, right, then I took the anti-venom. It bit me here on the right foot. The first time there was anti-venom in the city, only this time I didn't have it, that's why they sent me here. There is no medicine like that there, because we are worried about taking something and complicating it. I chose to come to the hospital right away, but the only thing I didn't have was the ambulance and there was no anti-venom there.

**** * Interview_11

I was going to a friend's house and had even invited another friend to come with me, but he went home. So I went on the trail, it’s made of clay, a clean path, I think it [the snake] was in the leaves. When I stopped at my friend's house, that was when my other friend passed by and I didn't see it. It looked like it was amended, I don't know. When I got close to this friend of mine, I asked him to go with me, it was when I stopped and I just felt my toe hurt, it was the snake, then I kicked it off and saw my bleeding toe, so I screamed for my friend who came back and he killed the snake and we brought it here. He also told us not to take it, but I decided to take it because it might be necessary for something, so we put it in a bag. I asked him to go with me to the accident and emergency riverside ambulance service station that was nearby. I got there, washed my foot, which hadn't swollen yet, but when I finished washing it swelled up. I washed my foot in the river to remove the clay. When I saw that I was a little swollen, I was afraid at the time and I felt that it hurt a lot. When I washed my foot, I felt my vision going dark, but it was the pain. I asked them at the accident and emergency riverside ambulance service station if they could bring me here to Manaus, because a snake had bitten me and I showed them the snake. At the time I signed a paper to confirm there is a parent/guardian in charge. So we arrived here at the port around eight-thirty in the morning. It was about forty minutes or so. Arriving at the port, I kept calling the accident and emergency riverside ambulance service – I called many times. I made several calls because it was almost nine o’clock. I took an Uber and asked the guy how much he would charge to bring me, so I came here straight away. I knew I had to come here because they had already said that snake bites are only treated here at the Tropical Foundation. I arrived here around nine or ten o’clock. I was worried about coming to the hospital. I just kind of tied my foot off with an old cloth - it was because I was told that I had to tie it up so venom would not spread. So I came with my foot tied up until I got here.

**** * Interview_12

I was working in the fields like this, clearing the field with the machete one close to the ground, which was when it struck, trying to bite me in the face and I defended myself with my hand. Then it bit me here on the little finger, so I grabbed my finger and kneaded it. I got home and found a scalpel. My finger was already all black from the venom, so I made a cut and squeezed it out a lot of the venom and I washed the bite with alcohol. I took that home remedy which is a liquid. In the package insert, it says to take a spoon in half a glass of water. I had a lot of milk, too. I waited, I stayed home for the afternoon. The accident was midday and I stayed there waiting until twenty to five. When I saw that I was spitting out pure blood, I sent for my son. I didn't want to come, but it was the only way, right? So I took our canoe with a small engine to Colonia Antônio Aleixo and from there, we got a friend with a car from to bring me here. I knew I had to come because this was the second time it has happened. The last time was more than twenty years ago. That time, it was on my foot, and the people who already knew told me to come here. This Tropical Foundation is more talked about than my grandmother's privates (laughs). Terra Nova to Colonia Antônio Aleixo takes about twenty-five minutes. We got there at twenty minutes to six. From there, we arrived here at the Tropical Foundation at seven o'clock. Right after the trauma I felt a lot of pain in my lower back and my head hurt, but I took my blood pressure medication and it went away. Here I started taking the medicines that made everything go away. Now only my pee that is still red with blood. I don't like going to the doctor’s, but as the problem was bad and my son insisted on me coming, we came, because to travel at night is quite dangerous.

**** * Interview_13

I was on the BR 174 highway at highway marker twenty-six when the accident happened. It is thirty minutes away, but with the track it takes about forty minutes because there are another eight kilometers to travel. At the time we were working, but we had already finished working, as we are lumber cutters. I went down to bathe in a small stream nearby and went to change clothes behind a tree in the woods. It was about five-thirty in the afternoon or so. When I stepped the snake, it attacked me. It was about five meters away from the lake where I had bathed. As we were there to saw wood for the owner there, the house was closed, so the only way was to bathe etc. in the woods. At the time, I only felt the bite. I didn't even think it was a snake, I thought it was a twig that had struck me. After, when I looked properly, I saw the blood coming out of the two holes from the fangs and I saw it with its tail wagging, I yelled to my father who came and killed it. My father was nearby too. At the time I didn't feel anything, just the scratch, but after a few seconds I felt my leg tremble and after I felt it hurt. To tell you the truth, my father brought me here, but we stopped at the Delphina Hospital first, thinking that they had a service there for this. They informed us that they did not have the necessary assistance and sent us here. At the time, I just washed the bite with water and got the car. About five and forty, we left where we were and went home to get some ID, we went to the Delphina Hospital and after we came to Tropical Foundation. We got here around seven-thirty. At the Delphina Hospital I don't remember the time, but it didn't take long because we didn't get past the reception. Here, [at the Tropical Foundation] I took the medication before to prepare me, and then the antivenom. I didn't feel anything afterwards.

**** * Interview_14

At the time I was clearing up at the family smallholding; I was close to home, which was like ten meters away. I was using a machete. There were some blocks of cement that we leave and pile up, so I went to lift one up with the machete and it was quick, it bit my left hand, right on my finger. I think I touched it but I didn't even feel it, I even thought, "Did it bite me?" When I looked it was on top of the block. It was a small twenty-centimeter-long snake. I just felt that burning, then I told my brother and he killed the snake and I said: "Let's go to the Tropical Foundation, which is the only place we know that takes care of that". We have a car, I just washed my hand and came straight here to the Tropical Foundation. This was the first time that this happened to someone from my family, but people say that the Tropical Foundation is the place that takes care of it. So I just got the car and came straight here. It was about twenty minutes to six when it bit me and we got here it was a little after seven. It took longer because of the traffic on Avenida Brasil. If it weren't for that, I would have arrived earlier. I didn't take anything, I just came straight here. There is no one nearby who teaches us about medicine, but as we have the car that is always there, we came straight away. You can come by small motor boat too, but it takes longer. Thank God that there is now this bridge that speeded things up for us a lot.

**** * Interview_15

At the time I was looking after the chickens, so I was so close to where the chickens are, and I saw my mother doing a job near the other chicken coop and I would go there with her to ask her to slow down a little, she is already old and tries too hard sometimes. I didn't see the snake, when I felt it was just the bite on my leg. It was on the path near the chicken coop. Maybe it would eat one of the chickens. I screamed for help, that's when my husband who was around came to help me. I looked and saw only the snake going through the bush. I said the snake had bitten me. I immediately felt a lot of pain and the leg swelled up. I told my mother to inside because there was a snake nearby and it could be dangerous. I immediately asked my husband to bring me to Manaus. We have a motorbike and he brought me on the back of the motorbike. The accident happened about seven o'clock and I got here around eight o'clock. I knew I had to come here because I had already brought my husband once because he had tuberculosis, and my daughter had already done a six-month internship here too. She is graduating in pharmacy. I didn't put anything on the bite, but I drank a lot of condensed milk (laughs) and I don't even like it. From home, to getting to the main road in BR, it takes about ten minutes on a motorcycle on the dirt road. What really took time was the traffic after the checkpoint that got congested because a truck had overturned. I arrived here on the sixth [of the month] at eight-ten in the morning and did the normal procedures. They discharged me on Saturday, but I had these blisters at the site of the snake bite and then I came back on the thirteenth and I'm here waiting for the surgeon to drain them.

**** * Interview_16

I was at a cousin's house, in a municipality called Pesqueira, in front of Manacapuru. I went there at about five o’clock to get some corn to make “mashed corn” on Sunday, December 8th. When I was in the field getting cobs of corn, walking through the corn plants, that's when I stepped on it and heard a squeaking noise by my legs, then I just saw the blood running and four holes. It stung and the four fangs got me at once, below and above. I immediately removed the laces from my shorts and tied my leg above the knee here, on the right leg. Then I came back and it was looking at me, shaking its rattle beside me. I left the corn, left everything. I got back to the house and they asked what snake it was, I said I don't know it because I don't live in the region. I just went for a walk at the house of my cousin who lives there. At the time I was alone! I went back to my cousin's house and there they gave me a pain medicine and put a hot egg on top to draw out the venom. They cooked it and put it on top. They cut the egg into slices, put it on top of the bite and tied it, without the shell. They tied it with a bandage. When it was about seven o'clock at night I couldn't take any more pain and I felt like my whole leg had started to go numb. I was still at my cousin's house and I could no longer stand or walk properly. They put me in a small motor boat that they had and transferred me to Manacapuru and I went to the hospital in Manacapuru. Once there, they started to give me medication and take the [necessary] steps. I asked them to transfer me to Manaus, they said they could transfer me, but the transport would not be their responsibility. There they gave the antivenom and did all the tests. They saw the amount of venom and I still have the exams here. They released me about four days later. I was admitted to there and took all the antivenom. Then I came home here to Manaus and they gave me medication to take, a pain pill and another that was an antibiotic. I was taking this pill for five days, that's when I disinfected everything, but this clot remained. Then it started to throb, throb and stop. I spent five more days at home, here in Manaus, taking the medication, but it didn't get better. I could see that the medicine I was taking was no longer working, I decided to come to Tropical on the 17^th^, on Wednesday. I knew I had to come here because we know that every snake bite has to come here to the Tropical Foundation, and this was the first time. They tied the egg on only for an hour because when I went to the hospital they took everything off. When I left there I didn't do anything because it was all too fast. There was a time that it was throbbing, it was painful and it was throbbing and it stopped, then it started to throb and the pain would not stop anymore, that's when I decided to come to the Tropical Foundation, where I did the blood tests and they did x-rays. The doctors kneaded my leg a lot, I felt hellish pain, but that's how it is. I started taking a potent antibiotic to see if it would clear the infection. I stayed at home because the people from the countryside kept saying: “It’s not venomous!”. I believed them because it’s something I didn’t know about, right? When I saw that everything was getting hard on my leg I said: “I want to go to the hospital!”. These things of tying the leg up and putting the egg on are things they believe in in the countryside, because people keep saying that so and so always do this when it happens. I don't even know the procedures and I didn't know anything. At the time of the bite, I just felt my leg going numb. I took a long time to come here too, because I thought here I would only be seen if I had a referral and the emergency room in Manacapuru didn’t give me one, so I kept thinking, will I go, will I come back, until I thought: “I will just like that! ” then they treated me here.

**** * Interview_17

At the time we were hunting and we went to some land that belongs to some people that we know from the small town where we live. We covered about twenty-five kilometers. I was in the woods, we get to the land and walk another ten kilometers into the woods, that's where the snake bit me. We were walking, so I went to the side of the trail and found some fruit, when I came back, clearing the way, it was when I cut a stick and just felt that shock on my leg, like a thump, then I ran forward to see it and it came like this and raised its head very close and I was looking and I said: “Wow! A snake bit me". I had the shotgun, so I shot it and killed it. Then I yelled to my mates and walked away pretty quickly. I caught up with them and it was about six o'clock in the evening. It was dark and they asked: “What is it?”. I said: “A snake bit me, man!” One replied: "So let's go, we are still far away". We were already putting things together and another mate arrived and he asked: “What was it?” and I said: “A snake bit me” and my leg started to hurt. I started feeling a numbness. I didn't vomit or bleed, it just made me very thirsty and dizzy. I didn’t put anything on it, only what he gave me which was a vine that he cut and gave me so I could drink that water. Then I just walked for a bit and I couldn't take it anymore. I wanted to steady myself with my leg, but I had no strength left. It hurt a lot when I stood on my leg so that it swelled up here in the knee. I walked a bit like this and said: “I can't take walking anymore”, then the lad said: “Carry him!”. My mate who was with me carried me. There was a moment when he got tired there, he lowered me and there was a time when I hopped with my left leg and came hopping and we came back like this. We had arrived by motorcycle, because from the city we come by motorcycle to the land. We arrived on the motorcycle on the man's land. We arrived at a clearing and I said: "I can't do this anymore", because it was hurting a lot, then he laid me down and said: "You have to put up with it, you will put up with it. I won't leave you here". Though he was very tired because he has a neck problem too. As he was too tired, he said “Now what?” and he would lift me up. That was about six o'clock on the nineteenth, a Wednesday or a Thursday. Then he came back with a man to help us. He said: "You go ahead and tell your son that the snake bit him". Then he said "it's good". Thankfully, by the time he left, his son was already on the way there, as he was going to hunt in the same place as we were. He was already in the middle of his trip, then his son came running. When I arrived I was sitting there feeling a lot of pain that when I got up it hurt. Then he arrived, I told him about the snake bite and he put me on his back and carried me, that's when we arrived at the home of a man who was already starting the bike, putting me on top and we left. We arrived in the city at half past seven. It was almost seven when we left the man's house. We came really fast, really quickly! Me on the back of the bike. Only he came with another guy behind him, holding me and with every bump, my God! It hurt so much! Every little shock that my foot took, you are crazy, it hurt too much. There at the hospital they gave me medicine to stop the pain, when they applied it here on the arm. It seems that the pain went up my leg and I screamed and the woman said: “Calm down, that's how it is!”. It started puffing up, just swelling up. We stayed there and she said: “You are going to stay here because there is no medicine against the snake venom here for you! We will only stay here giving you medicine to control the pain! ”. We stayed that night. The next day the antivenom came. I took the antivenom about seven o'clock at night the next day, they applied the antivenom and said that this antivenom was for the venom. When they applied it, I just doubled up on the stretcher and when I doubled up, I raised my head and felt bad, in agony and I told her that I was not well. She went and called the nurse and I felt like I was suffocating. They took me off the antivenom and gave me some medicine quickly and it passed. Then they said: "let's try again!". They waited and when an hour or so passed they tried again. When they tried again it came back stronger! As soon as it got into my vein, I didn't react well. I rejected it on the spot! It was closing up my throat and they put me on oxygen. They gave me oxygen and it passed! They said they weren't going to try anymore. They said they were going to send me to Manaus, that here they could treat me better. The next day, they were supposed to transfer me, but it rained too much so I stayed there. On the fourth day, I came. It was about eight in the morning. I came by plane! We landed at the airport and an ambulance was waiting for me. They brought me here and when we arrived we went straight into the treatment room. I was first given antiallergic medicine and then applied antivenom for the venom. At the first hospital, they had no antiallergic and tried to give me the antivenom straight. In my town, there was only antivenom and pain medication. I was putting a homemade remedy on top of the bite to reduce the swelling. I went to the hospital because of the swelling. Then I didn't put anything else on the bite. To Manaus, I went by plane, boat and another small motor boat. By small motor boat it takes an hour or a little more and the flight takes forty minutes. We took longer because the weather was kind of ugly. On the way, my mate also asked if he was supposed to stop off at my home to tell relatives, I said: "Man, go straight to the hospital because it really hurts". We went straight to the hospital and after he went to tell people at home. After the antivenom, I was also full of itchy red spots on my skin, but it wasn’t a lot.

**** * Interview_18

At the end of the year, we decided to take a few days off and go to the countryside, in Jandira, which is a lowland municipality and is close to Iranduba, about half an hour away. I went there with my family at the end of the year because we live here in Manaus. I went there just to spend the end of the year there. I went to do some weeding to clear the land, precisely to stop snakes coming in the house! I took the brushcutter and weeded. I was wearing boots while weeding and when I finished, I took the boots off and on the way back to the house, very close to home, that was when it bit me, about fifteen meters from home and I didn't see it. It must have been among the bushes because they were short, a foot high. It was small and I didn't see it well. As soon as it bit me, it ran away! It bit there on the toe of my left foot. At the time it just felt a needle going in and felt my hands go numb and tingle, then I was shaking all over. I went home and asked for help from my aunt to wash it with soap and water and put my foot in a bowl with ice water that was in the freezer and that's when I relieved the pain. It was already hurting enough. I had no symptoms other than pain and tingling in my hands. As we have all the resources there, the car was available and as soon as the snake bit me, I washed it and got in the car and came here. I knew I had to come here because, as I live in Manaus, we know which hospitals do the treatment. As it was the first time this happened, I came straight here. I left there at eleven in the morning and arrived here at noon. We came fast, at considerable speed by car. Here in the emergency room, when I arrived, it took a long time to get the antivenom because they drew blood and went to do the procedures there, it took a while, I took antiallergic medicine and after almost an hour and a half I took the antivenom. I took it in there and then they transferred me here. Only the pain didn’t stop! It hurts, a strong pain and relieves it. Only my toe is still swollen a little, but only now because yesterday my whole foot was swollen. Now that it has eased more. I'm already feeling normal, but even more today because we did the procedures quickly right? Because there was no traffic, just cross the bridge to make turn and come straight here.

**** * Interview_19

I went to get a small motorboat that I had left outside because I live in the woods. I live in Paraná de Terra Nova, in the São Francisco community which is upriver from the municipality of Careiro da Várzea. It was almost six o’clock and I took the small motor boat and on the way back I stepped on it, that was when it happened. It was about three hundred meters into the woods. It was just me and my wife there, she was carrying the paddle and I had the outboard engine on my shoulder. At the time I really felt the blow and I thought it was a wasp, I jumped with the engine on my shoulder and it bit me right there on my right foot. I jumped and was already lowering the speed of the small motorboat. The snake was small so I killed it and threw it away. I didn't even want to come, but I saw that it was going to be a problem. I went home and I lowered the engine to the ground, then I felt a lot of pain and my head also hurt a lot. The solution was to go to the riverside ambulance service. We got there but they had no gasoline. I came here via Puraquequara, I crossed the river with the ambulance of riverside emergency service. We bought gasoline and came. The riverside ambulance service only serves as a transport to bring us, there is no nurse or anything. It is right in front of the house on the pontoon about three hundred meters away. There was no gasoline there and the only way to pay for it out of your own pocket to buy gas, about a hundred reais of gasoline. So we jumped into riverside ambulance service with everything we had, we went up to the other pontoon [which is a floating gas station] and we put a hundred reais of gasoline and came. We came through Puraquequara. We arrived at the port of Puraquequara, called a sister-in-law who called an ambulance that was already there waiting for us and brought us straight here. That was about half an hour of transportation and we got here at around ten. I arrived feeling a lot of pain. I only put a cloth over it because of the wind that when it hit it hurt a lot. I washed it with soap too. Here I did all the procedures. This is the first time it has happened to me. You know, right, at the end of the year they give out that contract then it takes a long time to restart the work of the riverside ambulance service.

**** * Interview_20

I live in the interior, in the Sagrado Coração de Jesus community. At the time, I was cutting banana trees, removing the leaves. I work in agriculture, but I'm retired, I do it just to keep busy. When it happened it was the 10^th^, on a Friday at eight o'clock in the morning. When I arrived here, it was noon. I was cutting and I didn't see it, I stepped on it and it bit it me several times, I believe it bit me three times. At the time I took a shower and they brought me to Manaus. I didn’t put anything on it because each snake has a home remedy and this time, there was nothing close to home. That was the first time and I hope the last one right? (laughs). I waited for my cousin who lives in the village to bring me by small motorboat. I live there with my mom and my brother who lives nearby. At the time I killed her and left it there and I was like ten meters from home and my mother was in the yard too. I called my sister who lives in the village to ask for help. There is a cell phone and the signal is good. In about half an hour I went from the village to the Ceasa port by small motor boat. It was a really long time before he arrived to pick me up because, when he arrived, it was ten thirty. There is a small motor boat there from the riverside ambulance service nearby and its job is to go to the village, but from there to the village is another trip. Only they couldn't at the time because they were going to have a meeting. On the day there was a small motor boat, from there to the village, but from town to town there was no way to bring me. I called my cousin but he was late. I went on the small health service motor boat and from there I went to the village, but there in the village there was a meeting with the people from the small motor boat. This made it take even longer because there is a greater neglect of the riverside ambulance service, it only works for eight months and then it’s four months stopped. The mayor hires the riverside ambulance service for eight months and these four months are just meetings. Months of January, February, March and then in April he will pay until December. They only go there if we give them gas. Then I came here on the small health service motorboat to the port of Ceasa. My brother-in-law was driving and he didn't know where the hospital was. We just kept going round in circles, we ended up in Cidade Nova so I was lost with my brother-in-law driving, because he didn't know where the switchback was. My brother-in-law knew it was here because my cousin had already come here a month ago for snakebite, too. He knew then where it was just that he got lost to find a return here. I got here and it took a little while to take the antivenom. It took an hour. A doctor came to see me and took care of the medication. There are some remedies there in the interior that you take for this, but I didn't want to take it to make it more complicated. At the time, it felt like an ant bite and it didn't hurt at all. To tell you the truth, I didn't even want to come, but my sisters said: “Look at the example of your nephew who came before, he took his time coming and he almost lost his leg”. His bite was on Thursday and he only came here on Friday and his leg became very swollen and he almost died because the venom was already up to his knee. If I had come on time I would have arrived here at nine o’clock, an hour’s traveling.

**** * Interview_21

It was on the eleventh, about six-thirty in the evening and when I got here it was about ten-thirty at night. It took about four hours to arrive. I was walking because I was getting some wood, because I work with cattle and I was going to get some wood to do a job, so I was coming home like that, a couple of kilometers from home. I was coming with a friend and I think he stepped on it and it was scared and as I was behind I stepped on it too and it was soon attacking. The snake bit here on the heel of my right foot. When I felt it, I jumped and just saw it with its mouth open. So I killed it. My friend seemed to be more nervous than me. I said to him: “I don't even want to see this again!”, Then he threw it into the river, into the stream. I felt a slight sting at the time and bled just a little. I had been having a couple of beers and when I finished having the next one, I spilled the rest on top of the bite. I told my partner that I would come back home because there is help at home, but where I was going there wasn’t any. I live in Marimba. So I came back and the friend continued on his way. I told him to take care of the cattle and the farm and came back walking alone. Then my leg started locking like a sprain. It was just like a cramp. I was going to another house, but I went back to my house where my family was and my daughter-in-law told everyone that I had arrived. I told her to sort out some clean clothes because I was going to take a shower. I took a shower and washed my leg with a fresh bar of soap. Once, a nurse said that I had to wash with a soap I had never used before, so I remembered. I washed, got my clothes and asked for my ID, they asked what had happened, so I told them that the snake bitten me and that I was going to go to Manaus. That was at seven o'clock. They were worried and my mother, who has diabetes and heart problems, was already sick with worry. It was time for my daughter, my daughter-in-law and my son-in-law […]. I called the riverside ambulance service and after about half an hour they came. Where I live is the São Francisco do Paraná community in Terra Nova, and the small motor boat from the riverside ambulance service arrived in about half an hour. I did not pay for gas, but a cousin of mine paid for me, so when I get back I will have to give him money to pay gasoline because the riverside ambulance service didn’t have any. The riverside ambulance service there seems to have only eight months of contract that the mayor pays. Then there are four months without a riverside ambulance service because the end of December is the last payment and I will be honest, this riverside ambulance service that is there I have used it ten times and every time I have had to pay. It takes twenty liters of gasoline which costs one hundred and twenty reais because gasoline there is more expensive, it is five reais per liter. So, they brought me here via Puraquequara to Colonia Antônio Aleixo and from there I got a ride from a friend that I will also have to pay on the way back. It was about eight and I went there to the Emergency Service in Colonia, the Chapot. I went there and they gave me a referral here. I knew I had to come here, but from there they gave me a referral to come and it comes easier, right? I knew I had to come here because I had already brought a colleague of mine here and everything that is an insect bite is here, right? This here is the center. At the Chapot, they did the procedures, gave me injections and said I had to come here and I said “I know” then they put me in an ambulance and I got here around ten-thirty. It didn't take long, I just had an injection there and even I had some injections here in the shoulder that bled so much and then it turned purple and another one here in the butt that bled too. I think it's due to the snake's venom, you know? I bled because I could taste blood in my mouth too. When I arrived here they took blood, I was all purple and bled a lot and the nurse was even nervous because I was bleeding. I didn’t feel any pain, I felt it locking up my leg even without moving. From here down I can move the leg, but from here up I cannot move it at all.

**** * Interview_22

I was hunting, it was eight o'clock at night, I was close to the village hunting. I went out to kill a paca. I wanted to kill the paca and I wanted to return to my canoe, so it bit me. At the time I was with my friend. When I felt the bite I shouted for them to help me. My friend found the snake. I sat down and called him. He killed the snake, but we left it in the canoe with a small engine. My leg really hurt just from the bite on my toe. Only blood came out, but not much. We were in the canoe with the small engine. We took the canoe with the small engine and went back to the village. We had gone up the river, so we returned downriver to the village. So it was about fifteen minutes to the village. We walked to the canoe which was only fifty meters away. At the time I did not put anything on the bite, but in the village we put lemon and buriti oil on it and took some medicine, metamizole. We called a health care worker to get medicine. We have a shaman, he said that to put that stuff on it. We arrived at eight or nine, or around then. Coming downriver, we arrived at our base, it was eleven at night. We went by canoe with a small engine down the river. I came, my brother and the health care worker. From there, we arrived in Novo Airão and came here by car. We have a house in Novo Airão, too. Once there, we called a taxi from Novo Airão for people to come here and get us. We arrived in Novo Airão about four in the morning the next day. The delay is coming down the river. At the support house, she only gave me pain medication and to vomit, too, cleaned my foot and then we took the taxi here and we arrived here at half past six. I knew I had to come here because here in Manaus we have a support house, too. They already know what to do, so they tell us we have to come here. I just used lemon and buriti oil. I wanted to come quickly to help myself because this is dangerous, right?

**** * Interview_23

At the time I was working, planting passion fruit about a thousand meters from home around five o'clock in the afternoon. I was coming home and that was when it bit me. I believe it was under a thicket. At the time I was wearing boots, but it was when I went to untie the canoe that it bit my left hand. At the time it was just me and my brother, he was getting the cattle in, so I shouted to him and he came to get me. He was on a horse, he came by horse and at the time he took me home, then he went back there and killed the snake. It was about five o'clock in the afternoon, but I didn't put anything on the bite. We were soon getting ready to come. This was the first time that it happened to me. I went to Itacoatiara. My father took me to Itacoatiara. We went by canoe with a small engine, forty HP engine. We arrived in Itacoatiara and it was almost seven o'clock. My uncle was there waiting for me with his car and he left me there. Then they asked me what happened, they made me sling here on the arm. Then they gave me pills too. I don't even know what it was. It must have been for pain even though I had a headache. They said I didn't have the antivenom there and they brought me by ambulance. I arrived here at half past one or two o'clock the next day. Here I had the whole pack of antivenom. I had only milk at home, they said it was good, I had two glasses of milk. People say it's good. I don't even like to drink milk, but I had to take it to get well. I also had a glass of bitter coffee, coffee with water, bitter. It took me so long to arrive because of the distance. It took a long time at the hospital because I left the hospital at ten forty-five in the morning because they said they needed an authorization from the Mobile Emergency Service here to be able to make the transfer. That was the delay. The ambulance was already there, the delay was because since seven o'clock I had been waiting and left almost eleven o'clock waiting for the authorization to bring me. The ambulance trip was very quick from there to here.

**** * Interview_24

I went to my brother's house who had arrived from Itacoatira because he had bought gasoline and he takes it to sell there, so I had gone there with him, because my gas had run out. Around six o'clock, he invited me to dinner and I had dinner with him, then he told me that he had finished the bottles he had to sell and I left his house at about seven o'clock at night, only that I had forgotten to take my flashlight, then he said "Wait, I'm going to light the way for you". It is thirty meters from my brother's house to the port. He shone the flashlight over there so I could see the way. I could see the way there to the river where I had moored up, when I was about ten meters from reaching the canoe with the small engine, I felt something like a shock, then I kicked and shouted: “A snake bit me!”, then he went down to help me and I saw the snake hide inside the grass and then disappeared. I started to feel my head hurting and ten minutes later my leg started to hurt. My home was close to his. It is about five hundred meters, so he took me in the canoe with a small engine, moored at my house and went to call another guy to bring me here to the hospital and I stayed in the canoe with the small engine just waiting. At home, I took the home remedy, it is a liquid and I had two bottles. Since I take care of my brother's cattle, for the time they are in the fields, he bought me two bottles of this home remedy. We drink it mixed with a little in the water. So I took about ten ml and a little more water. At the site of the bite, I only used a cotton swab to remove that little blood that is on top. Then after half an hour the guy stopped by to pick me up in a canoe with a small engine. I arrived at the port and my brother-in-law was there with my sister and they took me to the hospital. It was already half past eight. What took longer was for me to be waiting for this guy with the canoe with a small engine, because he was searching for this boy and my brother was still waiting for someone to get him so he could bring me. There I waited for authorization from the Mobile Emergency Service to be able to bring me here. At that time, I left around ten or so, in the same ambulance as my acquaintance there, because I didn't have the antivenom there and they only gave me some medicines and I don't know what they were for. I arrived here at the Tropical Foundation at one o'clock in the morning. The ambulance trip was quick and I even blacked it out, I don't know if it was the effect of the medicine, I just know that when I woke up we were already here at the entrance and the nurse was calling my name out. I even threw up on the trip while I was in the canoe with a small engine, I threw up in the car to the hospital and in the ambulance from there to here. I was still spitting blood and my pee was yellow because I haven't drunk anything since yesterday.

**** * Interview_25

I was in the woods, and it was far away! It was more than fifteen kilometers from home, I think it was more because from ten o'clock in the morning until four-thirty in the afternoon people can walk a long way, right? That was the time I left. Walking all that way in the forest, I was lucky because when I arrived at the Careiro Castanho hospital they had the antivenom, so I had the injection, it was there that they gave it to me. Then they transferred me to the 28 de Agosto Hospital because the site of the snake bite was very swollen and the 28 de Agosto Hospital was where I came. I saw the snake, but I don't think I stepped on it! And it was already scared. After it bit I looked and saw it. I even thought it was a thorn. I had three colleagues with me! I was walking, they only carried me when there was only a kilometer left because my leg was locking and it hurt a lot, the whole leg and it was locking. It was getting stiff, and I couldn't move. I thought about returning home because I didn't take any medicine, nor did I put anything on it, I just left. I just came back. When I got home, a guy left me there at the ambulance station to get me dropped off in Castanho. He is a guy who works there up the dirt road, a colleague. He took me by van to a health clinic that is close to where I live, about five miles away. It was from the health center that they took me to the Careiro Castanho hospital. Lucky me, because the doctor said that the antivenom had arrived on Friday, it was about five-thirty to six in the afternoon. I took the antivenom on the same day and stayed overnight until about eight o'clock in the morning the next day and they transferred me here, to the 28 de Agosto Hospital. I came by ambulance from Careiro and crossed in the small motor boat, then there was another ambulance on this side that took me straight to the hospital. I think I got to the hospital 28 de Agosto about oneor two in the afternoon. There, the doctor checked the location of the bite and then transferred me here, to the Tropical Foundation. He went to see if the blood was circulating here in my leg and said that the blood was circulating normally and sent me here. I got here about four-thirty in the afternoon. Here, I only had antivenom and antibiotics. After that I just felt the pain in my leg, I didn't feel anything else. I can already move and fold my leg, which I couldn't do because it was rigid! The swelling has already gone from this part here, it’s gone down a lot. I was worried about leaving quickly because I knew how far away I was! I'm glad I managed to get here soon, I'm glad I was there at the time with my nephew and my two colleagues, Holy Mary! If I was alone there I would have arrived, but I would have suffered more (laughs). I took a home remedy that they say is good, but that was it.

**** * Interview_26

The accident happened at highway marker thirty-eight near Novo Airão. I went there and had to walk another twelve kilometers into the forest. I went with a guy who asked me to hunt for some uxí. The uxí is a small fruit that you have with coffee and manioc flour. We filled a shirt with the fruit and we were coming back, it was around three-thirty in the afternoon, that's when I felt it, on the way home. I think I hit a leaf and it got scared and then bit me. It bit my calf on my right leg and my leg was already feeling heavy, bleeding only in the place where it bit me. I walked back part of the way and the rest I ran back, then I took a friend’s canoe and went to another friend's house and from there I took the small motor boat to get to Manacapuru. From Manacapuru I came to Manaus. I just had some mastruz (wormseed) at my friend's house and put some over the spot where the snake bit, just mashed the mastruz and put it on top, then sat down and when I tried to get up I couldn't! There was a lot of pain and the leg was heavy. The small motor boat belonged to the boy who was there next to my friend's house and in that small motor boat I went to the port of Manacapuru. I arrived at the port around four-thirty in the afternoon and to reach the hospital, a boy offered me a motorcycle taxi and took me to the hospital for free. I went sidesaddle like a woman on a horse. My friend stayed there with the boat and then returned to his home. At the hospital in Manacapuru, they gave me the antivenom at about half past four in the afternoon and then they transferred me to Manaus. I arrived here in Manaus at about half past nine on Saturday night. They transferred me to the Tropical Foundation, but I don't know why they sent me here. I arrived here on the same day at half past nine. The road was very rough. It was almost two hours by ambulance, but I don't know why they took me by ambulance too, because, when I got to Manacapuru, it took almost an hour to give me an IV. They gave antivenom to me. Here, I was also given medicine for pain. The symptoms I had were pain and a swollen foot, now I'm just hopping. Before leaving my friend's house, I put the mastruz on the bite and only took it off when we arrived here in Manaus. I was worried about coming straight to the hospital because a snakebite is dangerous! The first time this happened to me was in two 2017, it seems that I was bitten on the other leg, on the toe, but I was just there in Manacapuru. There they gave me a pill and I stayed there. This mastruz, the people there say it helps, but it didn't solve anything (laughs). There on the trail I ran because my partner left me! He killed the snake and ran off towards the canoe (laughs). I stayed there following him, running but didn't know the way. He was running and I was running after him to not get lost. A right friend he is! But he's young, he's only sixteen. He must have got worried and just run (laughs). I just know that I don't even want to look in the direction of the dirt track back there, but we also walk around like that, just wearing sandals, but every day the people go there to get uxí and I had never been there before. When I went, this happened.

**** * Interview_27

I had gone to work to find wood, to make a shelter near the church. I got home from work tired, it was about five-thirty in the afternoon or so, then I took a shower at my mother-in-law's house and left my wife there. It was close to Careiro da Várzea, in the Divino Espírito Santo community. We had lunch and came, my wife was in front of me, she is five months pregnant. We climbed a ravine and there was a straight trail, but next to the bush there it was a little dirty, and can you believe it, the snake was right in the middle of the road in the open? But I didn't see it! When I saw it, it was already rattling and it was already biting me, and it was because I jumped back that it bit me, just once and I only took one fang. It was thick and big. It was about seven thirty at night already. My wife was in the front, you know, then I wanted to pass in front of her and when I passed four or five meters ahead the accident happened. Then I yelled, jumped and went after my mom to call the riverside ambulance service, which is very close because I live with my mom. I was only about ten feet away from home. Blood ran from the bite and after a minute I felt pain, a lot of pain. There is a riverside ambulance service there where we live, but because the mayor of Careiro da Várzea did not plan well, there was a shortage of gasoline for the boat. We had to take money out of our own pockets to be able to come. My mother who called and asked them to pick me up at the gas station and go there to pick me up because my house is about fifty meters from the river. To get to the small motor boat, they had to carry me, my father and my brother. That was at eight o'clock already. We got close to Careiro, passed by the beach and went around past the Terra Nova to be able to get to the port at Ceasa. Because there are pontoons and tree trunks in the middle of the river, he stopped to avoid them. Here we asked for an Uber, but it was because as he was a relative of ours I preferred it that way, because it was faster. I knew I had to come here because this had happened to my cousin too and he came to the Tropical Foundation, so I already knew what to do. When I arrived here it was already ten o’clock. I only washed the wound with soap and drank a few glasses of milk because people say it stops the venom. This riverside ambulance service is always there when you call them, they don't always have gasoline.

**** * Interview_28

I got home and after my shower I went out with my wife to go to church and we went out on our motorcycle. There in front of the church I parked the bike at the side of the road and the snake bit me. I was going to church. I had just arrived, I was going to park up the bike and that's when the snake bit me. The church is a kilometer from the house, on the highway marker fifty-eight of the main road, in Santa Luzia do Copaíba, in a community. It is about twenty-one kilometers from Lindóia, the nearest city. Access is by car and motorbike to the city. After the snake bite, we still went to church and attended the service anyway. After the service the pastor said: "I'm going to call the ambulance", then he called and the ambulance went to the house to pick me up. I had already come home. I came back riding the bike because we were with our two children. We have a nine year old girl and a one year old boy. We arrived home and shortly afterwards the ambulance arrived to take us to Itacoatiara. It arrived around eight-thirty and we arrived in Itacoatiara at nine o'clock at night. When we called, they already told us that there was no antivenom. It is fifty-eight kilometers away from my home. Her sister who lives in Itacoatiara who went to pick up the children there at the hospital, as they had gone with us. My wife is here with me and our children are with her sister. This all happened on Wednesday and there was only one metamizol tablet there, but I felt only intense pain, caused by local inflammation, giving a slight throb. They sent me straight here because there was no antivenom to take. We left Itacoatiara at twelve-thirty the next day to come to Manaus. I think it took a while because the ambulance was coming from other hospitals, leaving other patients, because four patients came with us. She left there with four and left two in another hospital and brought two here to the Tropical Foundation. I arrived here around four in the afternoon. The ambulance came down the road, but there was a hole because they did a repair, but it was badly done and the ambulance came like a rocking horse and couldn't go very fast. Here the service was better. I didn't want to come to the hospital! My foot was not swollen and I even said that it hadn't bitten me (laughs). The pastor was concerned and called. My wife looked at my foot and saw that it was bleeding and got worried. Every time I go in shoes, but the day I went in sandals, just on that day, I stepped on it and it bit me. I had arrived at the church and I just went back to park the bike. I did not take anything, nor did I put anything on it, because they say that when the animal bites you can’t put anything on it, nor drink water because the venom spreads right? My grandparents said that. It took longer because this ambulance that had these trips before us. The thing they say is that there is no antivenom there, you know, but it is more expensive or easier to have the antivenom there than to bring patients here! And Itacoatiara is right there [near home].

**** * Interview_29

I was expelled from school a year ago because I was very quiet (laughs) so I didn't study anymore, but I'm going to work now with my dad in the jungle.

I had been working, making ‘picadão’ for three days in a canoe with a small motor going up the Aripoanã River, then I just felt the strike here on my left foot about 9 am. At the time I didn't feel anything, but my cousin carried me home, to the shack and when we arrived at the house we got ready and we already left for the city. I took the home remedy which is a medicine that the guy takes against the venom. We drink it with water in a glass, I took it and went to get a canoe with a small motor. I went back to Novo Aripuanã and arrived at seven o'clock the next day, which is faster because it is going down the river. There were two engines running to get down the river, so it's faster. We went straight to the hospital where there was the ambulance that came to pick me up by the port. I arrived at the hospital at about nine o'clock. There I got the drip because I didn't have the medicine for the venom. They said at the time they didn't have it, so we just spent a day waiting there and took the plane to Manaus. The accident happened on Sunday, I spent the Monday at another hospital and on Tuesday I came to Manaus, where there was an ambulance at the airport that brought us here. It was already afternoon, later than noon when I arrived here at Tropical. Only here did I get the antivenom. From there you can come by boat, but it's three days by boat and by land it takes longer, I don't even know how you come by land. At home I took only the home remedy, nothing else and they also washed my foot with saline. I didn't have any other reaction here, but it's better because I didn't even move my leg like that, now it's already less swollen, because it was swollen from the thigh to the foot. The difficulty was the distance because there is no antivenom where I live. At the time of the bite I was with dad and other people working. When I came here, dad stayed and my sister came with me, she is at auntie’s now doing the laundry.

**** * Interview_30

I work in the fields, but it has been eleven years since I had an accident and hurt my back, in my spine and I was unable to do heavy work. I'm there in the fields because I like it. I am diabetic, I have a problem with high blood pressure and a heart problem, I have a catheterization in my arm, so I am there in this area that I bought, working away. I receive a pension due to this health problem. I live in Pau-Rosa at highway marker twenty-seven. On the day of the accident I was out getting sticks to make a chicken coop. When it was time to come back for lunch, about twenty-five meters away the snake bit me. It was half past noon and I had another twenty-five meters to get home. It was going along a narrow path and when I felt it, it was as if some thorns had passed over my leg, with a warm body and when I had gone about five steps further I felt my leg sting and then I sensed that it was not thorns. I looked at the leg and it was bleeding. I came back slowly, it was there curled up to strike again, then I took the machete, but when I raised my arm it came at my hand, but I twisted my body and it fell to the side. As I have already been bitten by seven snakes in my city before that bite, there in Envira, which is after Eirunepé, I already knew what it was like, except that in these other accidents I never went to any hospital. I was cured at home with medicine from the forest. I used a home remedy that I tried to find here in Manaus and never did. It is a branch that looks like a leaf which is used for a home remedy. The leaf is long and narrow. There are white and purple ones. Both will do, but purple is the best. At the time, in fact, my mom would take it from the rubber plantation, beat and squeeze it, get the juice more or less a glass of juice. The bagasse she put on top of the bite and the juice of the juice we drank with water in a glass. So what we drank worked against the venom, it stopped the effect of the venom. My mother sometimes gave us it according to the time we got home. For example: let's say that at three o'clock in the afternoon I would take it and then she would give it to me at midnight and around dawn the day she would give me another one and that was enough. This was indigenous knowledge because she comes from a Peruvian family, her mother was the daughter of a legitimate Peruvian and my grandmother passed it on to my mother and today it has passed on to us too. Here in Manaus I struggle to find it, and I can't find it. People don't even know what it is. It is a very wonderful plant. Continuing the story of the snake, as it jumped and failed to bite me I let it go because the last one that bit me I lost my sight at the time, I had very blurred vision so I couldn't see anything, so I thought that this could happen again and I came home, but nothing happened. I was not feeling anything, just a burning sensation, a burning sensation at the bite site. I got home and she said that our friend passed by five minutes ago by car and I was in front of a guy over there who has a motorcycle, I told her to ask him to catch her up on his motorcycle and bring her back. He was very close and in about fifteen minutes she was back with us. I was already prepared to leave and this guy's family stayed there taking care of the things my wife was doing. We arrived here at about two-ten in the morning. I knew I had to come here because I had brought my son a year ago, also due to snakebite. He stayed in this room next door. My son took a lot longer than I did because we went after transportation. I had nothing else, just the pus that had on my leg that started to hurt and they had this surgery done to remove the pus, right? A drain was made. I'm glad I had this friend of mine nearby because it's very difficult. If a government or mayor would listen to our needs and could put at least one first aid outpost there for us, it would be good. At these times, placing an outpost that remains open until five o'clock would also be good, because when it is two o'clock in the afternoon the outpost is closed. From my house to the outpost it is another thirteen kilometers and still a hell of a walk (laughs).
