# Supplementary material for: A painful journey to antivenom: The therapeutic itinerary of snakebite patients in the Brazilian Amazon (The QUALISnake Study)
Source: PLoS Negl Trop Dis. 2021 Mar 4;15(3):e0009245. doi: 10.1371/journal.pntd.0009245 (PMC7963098; doi:10.1371/journal.pntd.0009245)
Supplement: Attachment — Submitted filename: Letter TI.docx [file pntd.0009245.s002.docx]

**Responses to Reviewer's Questions**

Reviewer #1: This is a qualitative study, based on the in-depth interviews for describing the therapeutic itineraries. The sample size has been defined based on the data saturation.

Reviewer #2: (No Response)

Reviewer #3: The study population was representative of opinion from several different municipalities. The cross-sectional, qualitative methodology was appropriate to the objectives, as was the data analysis. The rationale for this study and its methodology was clearly described.

The sample size seemed low to me - especially given the number of variables assessed (particularly in Figure 5).

RESPONSE: Thanks for the comment. We understand the reviewer's concern. However, it must be remembered that this is a qualitative study, in which the main product is the analysis of patients' discourses. In this case, the main outcome is not to answer about the difference in the frequency of variables, which would only be possible with another type of design. Thus, the results presented for the quantitative variables are exploratory, and serve only to raise hypotheses for future studies, with appropriate methodologies, including sample calculations suitable for each case. In this study, we used the saturation methodology, in which saturation can be simply defined as data satisfaction, that is, when the researcher reaches a point where no new information is obtained from further data. It is a sampling method very often and traditionally used in qualitative studies. The number of patients analyzed in this study was similar or greater than studies of the same nature.

I would like to have understood why children under 18 years were excluded - this cohort is often the most frequently bitten by snakes and typically suffer more, and more rapidly, than adults. A brief rationale for this decision would be valuable.

RESPONSE: This is a very important question. This was a concern of the research group as well: to exclude the population of minors, which is less affected than adults of working age in the Amazon, but who in the case of complications, will suffer from the possible sequelae for a long time. After discussion between the research group with a specialist in qualitative research based on interviews of the participants, it was clear to the group that the type of questionnaire to be used for dependent minors should be different. In addition, there are still many doubts about the methodology to be used in qualitative studies with children and adolescents, and in these cases, parents or guardians would have to be interviewed in advance, to obtain more complete and reliable information. Again, it would be more of a collection instrument to be used and the final integration of the data would be complex and questionable. For the future, the group is designing specific studies with these age groups, with the help of psychologists and educators, in order to ensure that correct methods are employed.

I would like to have understood better the influence of schooling, access to snakebite care information (media, lay knowledge) had on perceptions of conventional hospital care and the decisions made to access it.

RESPONSE: In our study, schooling was slightly higher in the group with early medical assistance. We did not specifically investigate the effect of this variable on the time to medical assistance. However, we believe that the more educated participants tend to be more informed about the procedures to be adopted after the bite, have more financial resources for transportation to the hospital, and may have greater interaction and ability to articulate in the community, facilitating access to health system.

This information was added in Discussion *(Lines 711-717).*

How were bites ascribed so robustly to Bothrops atrox (100%)? Was some kind of diagnostics available? A brief explanation would be valuable.

RESPONSE: Thanks for the comment. Actually, we presente as *Bothrops* envenomations (genus, not species). *Bothrops* diagnosis in our study was based on clinical-epidemiological criteria. The positive predictive value of *Bothrops atrox* envenomations clinical-epidemiological diagnosis in this region reaches 97.8–100% using an monoclonal antibodies immunoassay as reference [1,2], due to the high prevalence (~90%) of the *Bothrops* genus perpetrating envenomations [3].

We added the diagnostic method in the table legend and with that we believe that we solved the reviewer's concern, which is totally justified.

References:

1. Pardal PPO, Souza SM, Monteiro MRCC, Fan HW, Cardoso JLC, França FOS, et al. Clinical trial of two antivenoms for the treatment of *Bothrops* and *Lachesis* bites in the north eastern Amazon region of Brazil. Trans R Soc Trop Med Hyg. 2004;98: 28–42.

2. Sachett JAG, da Silva IM, Alves EC, Oliveira SS, Sampaio VS, do Vale FF, et al. Poor efficacy of preemptive amoxicillin clavulanate for preventing secondary infection from *Bothrops* snakebites in the Brazilian Amazon: A randomized controlled clinical trial. PLoS Negl Trop Dis. 2017;11: e0005745.

3. Monteiro WM, Contreras-Bernal JC, Bisneto PF, Sachett J, Mendonça da Silva I, Lacerda M, Guimarães da Costa A, Val F, Brasileiro L, Sartim MA, Silva-de-Oliveira S, Bernarde PS, Kaefer IL, Grazziotin FG, Wen FH, Moura-da-Silva AM. *Bothrops atrox*, the most important snake involved in human envenomings in the amazon: How venomics contributes to the knowledge of snake biology and clinical toxinology. Toxicon X. 2020;6:100037.

The authors and their team deserve congratulations for this physically difficult study and one requiring considerable sensitivity to acquire the detail obtained. It would be informative for other studies being planned to learn whether they experienced issues over participant expectations following the interviews - financial assistance, travel assistance, treatment, etc? if so, how were these managed?

RESPONSE: Many thanks for the comment. Indeed, some patients in this study reported financial difficulties that stemmed from the acquisition of fuel for cars or boats (either their own or borrowed) expenses with taxis, medicines and food during the journey. However, the participants' speeches did not demonstrate expectations about financial compensation of any kind. Treatment for snakebites in the hospital is offered free of charge in Brazil. In the Discussion section, we added the the following sentences/texts:

*“Regarding affordability, antivenoms in Brazil are distributed free of charge to patients.” (Lines 575-576).*

*“One factor that can affect the accessibility to antivenom is the difficulty that the victim encounters in obtaining care is related to the costs of travel, procedures and obtaining pre- and post-hospitalization medications, as well as the loss in income due to time of work. Some patients in this study reported financial difficulties that stemmed from the acquisition of fuel for cars or boats (either their own or borrowed) expenses with taxis, medicines and food during the journey. In the Brazilian Amazon, the total cost of snakebite was estimated to be almost US$8 million in 2015, with US$1,539,518.62 attributed to the loss of productivity due to absence from work and US$268,914.18 being the cost from the patients’ perspective [69]. Since victims of snakebites are generally economically vulnerable, reducing this barrier of distance by improving or providing transport systems or reducing costs for the patients through the use of cash rewards or refunds [70], which may have a positive impact on snakebite victims’ accessibility to health care.” (Lines 581-593).*

Reviewer #1: The four themes identified are appropriate and are and described adequately. They are adequately supported with the quotes. They are well-supported with the figures and tables.

RESPONSE: Thanks for the comment.

Reviewer #2: (No Response)

Reviewer #3: The analysis was performed as planned and the results were clearly and informatively presented.

RESPONSE: Thanks for the comment.

I think some of the figures (at least those in the manuscript available to me) lacked the resolution required for publication - for editor's attention.

RESPONSE: Thanks for the comment. We went back to figures to check the resolution and they all obeyed the journal's rules.

I'd have been interested in an explanation why the elderly cohort accessed healthcare sooner than the other groups.

RESPONSE: In the group with early medical assistance, there was a predominance of participants over 40 years of age; elderly (≥60 years) were present only in this group. The quotes indicate that older people sought medical help more readily because of their own previous experience or with family members bitten by snakes. In addition, these individuals often have a higher income than other members of the community, due to retirement amounts by age.

This information was added in Discussion *(Lines 717-722).*

--------------------

Reviewer #1: The six conclusions arrived are well-justified and the limitations are adequately highlighted.

RESPONSE: Thanks for the comment.

Reviewer #2: (No Response)

Reviewer #3: This is a very valuable manuscript and addresses a paucity of research on the (i) perceptions and prior knowledge of rural snakebite victims, (ii) how this influences their healthcare-access decision making and (iii) the substantial barriers they face in accessing healthcare when they chose to do so. The very fragmented travel routes to healthcare was a very important delivery of this study. A cost-implication analysis would have been a valuable addition (but you can't do everything).

RESPONSE: Thanks for the comment. Anyway, we have published previously a cost analysis of snakebites treatment in the Amazon, including an analysis from the patient’s perspective (Please see: Magalhães SFV, Peixoto HM, de Almeida Gonçalves Sachett J, Oliveira SS, Alves EC, Dos Santos Ibiapina HN, et al. Snakebite envenomation in the Brazilian Amazon: a cost-of-illness study. Trans R Soc Trop Med Hyg. 2020;114: 635–642. doi:10.1093/trstmh/traa005).

As in the few other studies of this genre, this research identifies that victims often do understand bite severity and that this influences decision as to whether, or not, to seek hospital care. It was very instructive to learn that (i) that snakebite victim ITs often bypassed more local hospitals in favour of referral hospitals and (ii) that the vast majority of local health facilities had adequate stocks of antivenom.

RESPONSE: Thanks for the comment.

--------------------

Reviewer #1: Terminology: ‘snake poisoning’ is a wrong term, it must be replaced with “snake envenoming”

RESPONSE: Changed accordingly.

Line 354: remove additional comma

RESPONSE: Removed accordingly.

Throughout the manuscript (in the text and the tables), the percentages of patients have been expressed with the precision of one decimal point. This study includes only 30 (<100) participants. Therefore, it is not accurate to express percentages using decimal points – please remove the decimal values.

RESPONSE: Change was made accordingly.

After each quote, it would be appropriate to include the age, sex and ethnic background of the participant.

e.g.: ““We went after a more powerful boat you know, to take me to Manacapuru, but the owners there didn't want to take me at that time, afraid of the drug traffickers on the Solimões River, right?” (Participant 6: a 36 year old indigenous male)

RESPONSE: Informations were added accordingly.

Reviewer #2: (No Response)

Reviewer #3: While this is a very well written, referenced and balanced manuscript, some minor issues need attentions:
RESPONSE: The new version of the manuscript was revised by a native English speaker with experience in scientific writing.

The abstract and Author summary far exceed journal guidelines.

RESPONSE: Text of Abstract and Author Summary was reduced in size accordingly.

Numerous spelling mistakes need editorial attention.

RESPONSE: The new version of the manuscript was revised by a native English speaker with experience in scientific writing.

--------------------

Reviewer #1: The study titled “painful journey to antivenom: the therapeutic itinerary of snakebite patients in the Brazilian Amazon (The QUALISnake Study)” by Cristino et. al. describes the therapeutic itineraries of snakebite victims in Brazillian Amazon. Understanding the socio-epidemiology of snakebite, specially on the treatment seeking aspects in most vulnerable communities is of highest importance in confronting this neglected tropical disease. As such, the most important approach would be to use qualitative approach, which is very rare among snakebite literature. In this excellent manuscript, the authors have taken a qualitative approach, based on the in-depth interviews, in describing the therapeutic itineraries. I much enjoyed reading this manuscript.

RESPONSE: Many thanks for the comment.

Reviewer #2:

1. Snakebite is a neglected fatal disease among the poorest and among isolated communities. Anti-venom is the only hope for saving life at snake envenoming. According to the authors, the objective of this study is to identify the barriers in access to treatment and recommend improvements need in policies/strategies to handle the situation, by analyzing the therapeutic itineraries of snakebite victims. This area of research is important and relevant to the scope of the Plos-NTD.  Thanks for researchers for selecting this topic to bring the attention of the world to understand the treatment access to snakebites in Amazon region.

RESPONSE: Many thanks for the comment.

2.      This is a qualitative research under four thematic topics (1.Activity during exposure to bite, 2. Home remedies, 3.Knowledge of the snakebite injury and treatments, 4. Itinerary bite to treatment). Study sample consists of 30 study participants. From the statistical point of view, study design make sense to me. The objective of the study line 237 is clear to me but I feel researchers should give further attention in text to form the research question more clearer.

RESPONSE: To clarify this point, the sentence was changed to:

“The objective of this study is to describe the itinerary from the moment of the snakebite to the patient’s admission to the reference unit where the antivenom was administered, located in Manaus, in the Brazilian Amazon.” *(Lines 201-203).*

3.  I am not fully agree with the use of term “therapeutic itinerary (TI)” in this article because it is more appropriate for an illness that may not much time sensitive.  Snakebite envenoming is a life threating injury need immediate medical attention within few hours of the incidents unless or otherwise it is a dry bite or non-venomous animal bite. The all therapeutics described in this manuscript are temporary home remedies.  I suggest, sometimes “Itinerary from bite to treatment” would be more meaningful in this context than TI.

RESPONSE: A therapeutic itinerary (TI) may be defined as is the set of processes by which individuals or groups choose, evaluate and adhere to certain forms of treatment [1]. The concept of TI includes the search for different healthcare and describes and analyzes individual and socio-cultural health practices to solve the patient’s illnesses [22]. Then, by definition the study of TI need to include all health care subsystems [3]:

a) The popular subsystem, which comprises the non-specialized field and is most often related to subjective interpretations of the self-care.
b) The folk subsystem, which is based on spiritual rituals and practices carried out by shamans using medicinal plants and prayers. This still constitutes the main component of healthcare for some populations.
c) The professional component of the system, in which official medicine including antivenom treatment is included.

These definitions and additional informations were clarified in the Introduction. *(Lines 141-165).*

References:

1.	Demétrio F, Santana ER, Pereira-Santos M. The Therapeutic Itinerary in Brazil: systematic review and meta- synthesis from the health negative and positive conceptions of health. Saúde em Debate. 2019;43: 204–221. doi:10.1590/0103-11042019s716

2.	Gerhardt TE. Therapeutical itineraries in poverty situations: Diversity and plurality. Cad Saude Publica. 2006;22: 2449–2463.

3.	Kleinman A. Concepts and a model for the comparison of medical systems as cultural systems. CONCEPTS A Model Comp Med Syst AS Cult Syst. 1978;12: 85–93. doi:10.1016/0160-7987(78)90014-5

4.      Line 152-153; Sentence needs to improve and “preferably in the first hours after the bite” should be specific. May studies suggest within first 6 hours of the bite (with a reference).

RESPONSE: Change was made accordingly.

5.      Line 172-173;  I guess “delay for treatment” would give a better meaning to the phrase instead ‘late medical care’.

RESPONSE: In the new version of the manuscript, this part of the text was changed to decrease the size of the Introduction.

6.      Introduction of this article is very long to me to grasp the story line.  Authors can consider, details given in lines 188-227 to further summarized or move to the discussion or present in an appendix.

RESPONSE: In the new version of the manuscript, ~500 words (2 full paragraphs) of the Introduction were cut off.

7.      Line 284; ‘late medical attention’ or ‘delay for access to treatment’ is sometimes more meaningful than ‘late medical assistance’?

RESPONSE: Change was made accordingly in this line and in the corresponding Table.

8.      Line 293-294; need more clarity of ‘what kinds of inconsistencies ’ in this statement.

RESPONSE: Sentence was changed accordingly.

9.      Is it possible to elaborate the categories in table 3 to add  ‘Distance traveled (KM) reach to the hospital ’, ‘mode of transport’, ‘alternative treatments before deciding hospital option ’ etc? by summarizing the numbers that have been presented in Fig 2, 3 and 5.

RESPONSE: We added frequencies for those variables in the Figure 5B. Variables’ frequencies between patients who arrived delayed to the hospital and those who had early medical assistance.

List of variables:

1. Feeling of safety at home and work places; 2. Absence of help from other people, means of transport or communication; 3. Feeling of isolation in the place of residence or work; 4. Lack of knowledge of the route to take to reach the hospital; 5. Use of traditional treatments; 6. Exposure to snakebite in daily activities; 7. Perception of the seriousness of the situation experienced; 8. Timely identification of warning signs/symptoms; 9. ≥200 km to reach hospital; 10. Previous experience with snakebites; 11. ≥3 means of transport needed; 12. River transport needed; 13. Waiting for transportation ≥1 hour over the itinerary. Variables numbered 1 to 8 emerged from the interviews’ satements and variables numbered 9 to 13 derived from the physical itinerary analysis.

10.     I understand authors are not native English speakers. The English language use in Lines 350- 550 is confusing not well written.

RESPONSE: RESPONSE: The new version of the manuscript was revised by a native English speaker with experience in scientific writing.

11.     Line 577; 1 doctor per 5,000 would be more appropriate. You can’t have a 0.2 doctors.

RESPONSE: Change was made accordingly.

12.     Does Brazil has a public funded emergency ambulance system like 911 in US, 118 in India? Snakebite apps like in India? Those save many lives ( [https://doi.org/10.1016/S2352-3018(18)30007-9](https://doi.org/10.1016/S2352-3018(18)30007-9))

RESPONSE: Excellent question. Yes, Brazil has an emergency ambulance system (190), but the coverage deste sistema se dá basicamente para áreas urbanas e rurais na proximidade. Algumas iniciativas de snakebite apps existem, mas nunca foram colocadas em prática de forma contínua, infelizmente. De qualquer forma, as ambulâncias do 190 em geral não chegam nas localidades remotas onde ocorrem a maior parte dos acidentes ofídicos. Além disso, em muitas dessas localidades, não existe sinal telefônico ou os pacientes não teriam telefones para fazer ligações para o sistema de saúde.

13.     I had some limitations in this review. I am sorry my inability to check some of the non-English references, as I am not familiar in languages other than in English.

RESPONSE: The new version of the manuscript was revised by a native English speaker with experience in scientific writing.

Reviewer #3: I think this study is an outstanding contribution to our knowledge and one that the should help inform remedial interventions implemented by national, regional, NGO and community group stakeholders.

RESPONSE: Thanks for the comment.
